# Indexing Natural Products for Their Potential Anti-Diabetic Activity: Filtering and Mapping Discriminative Physicochemical Properties

**DOI:** 10.3390/molecules22091563

**Published:** 2017-09-17

**Authors:** Mouhammad Zeidan, Mahmoud Rayan, Nuha Zeidan, Mizied Falah, Anwar Rayan

**Affiliations:** 1 Molecular Genetics and Virology Laboratory, QRC-Qasemi Research Center, Al-Qasemi Academic College, P.O. Box 124, Baka EL-Garbiah 30100, Israel; mouhammad.zeidan7@gmail.com; 2 Institute of Applied Research-Galilee Society, P.O. Box 437, Shefa-Amr 20200, Israel; mahmoud_ryan@hotmail.com; 3 Clalit Health Service, Diet and Nutrition Unit, P.O. Box 789, Arara 30026, Israel; nuhayz@gmail.com; 4 Eliachar Research Laboratory, Galilee Medical Center, P.O. Box 21, Nahariya 22100, Israel; MiziedF@gmc.gov.il; 5 Faculty of Medicine in the Galilee, Bar-Ilan University, Ramat Gan 52900, Israel; 6 Drug Discovery Informatics Laboratory, QRC-Qasemi Research Center, Al-Qasemi Academic College, P.O. Box 124, Baka EL-Garbiah 30100, Israel

**Keywords:** diabetes mellitus, anti-diabetic drugs, drugs analysis, ligand-based screening approach, bioactivity index

## Abstract

Diabetes mellitus (DM) poses a major health problem, for which there is an unmet need to develop novel drugs. The application of in silico techniques and optimization algorithms is instrumental to achieving this goal. A set of 97 approved anti-diabetic drugs, representing the active domain, and a set of 2892 natural products, representing the inactive domain, were used to construct predictive models and to index anti-diabetic bioactivity. Our recently-developed approach of ‘iterative stochastic elimination’ was utilized. This article describes a highly discriminative and robust model, with an area under the curve above 0.96. Using the indexing model and a mix ratio of 1:1000 (active/inactive), 65% of the anti-diabetic drugs in the sample were captured in the top 1% of the screened compounds, compared to 1% in the random model. Some of the natural products that scored highly as potential anti-diabetic drug candidates are disclosed. One of those natural products is caffeine, which is noted in the scientific literature as having the capability to decrease blood glucose levels. The other nine phytochemicals await evaluation in a wet lab for their anti-diabetic activity. The indexing model proposed herein is useful for the virtual screening of large chemical databases and for the construction of anti-diabetes focused libraries.

## 1. Introduction

Diabetes mellitus, an expanding pandemic worldwide, is expected [1,2] to affect more than 640 million individuals by 2040 [3]. Diabetes mellitus type 2 (T2DM), one of three types of diabetes [1,4], accounts for more than 90% of all cases now diagnosed at any age, even in children [5]. T2DM is characterized by gradually progressive insulin resistance in various body tissues (liver, muscle and adipose), or failure in islet β-cells, or both [6,7,8], leading to the development of chronic hyperglycemia [9,10]. In T2DM-affected individuals, other cardiovascular risk factors (hypertension, dyslipidemia and obesity) are abundantly present [4,11,12,13]. If uncontrolled, T2DM leads to nephropathy, neuropathy, retinopathy, and amputations and poses a high risk of cardiovascular and cerebro-vascular events [4,14,15,16,17,18]. Due to the multifactorial nature of T2DM, no single anti-hyperglycemic agent can correct all abnormalities [10]. At this point in time, glucose control is the major focus of the management of T2DM, along with the management of cardiovascular risk factors, which includes reducing the duration of the disease, smoking cessation, the adoption of healthy lifestyle habits, blood pressure control, lipid management, patient adherence and resources and, in some cases, antiplatelet therapy [19,20,21,22,23,24,25,26,27,28,29,30]. The complexities of treatment entail enormous health, economic and social burdens. In practice, soon after diagnosis, metformin ‘monotherapy’ or ‘dual therapy’ is initiated, consisting of one of six treatment options: (1) sulfonylurea; (2) thiazolidinediones (TZD); (3) dipeptidyl peptidase 4 inhibitors (DPP-4 inhibitor); (4) selective sodium glucose cotransporter 2 inhibitor (SGLT2 inhibitor); (5) glucagon-like peptide 1 receptor agonists (GLP-1 receptor agonist); or (6) basal insulin. When the glycated hemoglobin (HbA_1c_) target is not achieved, ‘triple therapy’ combinations that do not include metformin may also be considered [19]. However, there is a limited potential with the use of any of the available drugs in patients with T2DM because balancing the glucose-lowering efficacy, the side-effect profiles, the anticipation of additional benefits, cost and other practical aspects of care is difficult. Moreover, data on the side effects of most of the possible drug combinations are lacking. Hence, the quest for new compounds that can complement current therapies for T2DM and its comorbidities is expanding [31,32]. Notably, natural products offer many potential mechanisms of actions for improving glucose homeostasis, which could reduce and/or abolish diabetic complications [32,33,34]. Since drug discovery and development involve time-consuming and expensive processes, computer methodologies are utilized to shorten the time span of drug development and to reduce costs, and in silico techniques may contribute to the identification of new lead compounds and to the optimization of drugs in clinical use [35].

In efforts to detect novel bioactive ligands, ligand-based techniques, including properties-based and pharmacophore-based tools, are being used more and more for modeling the bioactivity of molecules and for the virtual screening of large chemical databases [35,36,37,38]. Ligand-based modeling tools use optimization algorithms such as Monte Carlo simulations (MCs), simulated annealing (SA) [39], genetic algorithms (Gas) [40], neural networks (NNs) [41], support vector machines (SVM) [42], the k-nearest neighbor algorithm (kNN) [43,44], Bayesian classifiers and some combinations thereof (Monte Carlo/ simulated annealing algorithm, MCSA) [45,46,47,48,49]. Distinguishing between active and inactive ligands that are useful for treating a certain disease may be accomplished by using sets of active and inactive chemicals and certain optimization techniques [50,51,52]. Such techniques presume that bioactive ligands have common features that are not easily recognizable if only a small number of bioactive ligands are tested. Therefore, if a database contains a larger number of active/inactive ligands, drawing more significant and robust conclusions about the properties of these chemicals is achievable. In addition, it is essential to include in the training set of inactive chemicals, chemicals that cover the same property space as those in the screened chemical databases. In this way, analyses of sets of active/inactive ligands may shed light on characteristics leading to the bioactivity of active ligands. Due to the large number of descriptors taken into consideration during the modeling process, special optimization techniques that can overcome the limitations of the combinatorial nature of the problem of molecular bioactivity indexing are required. To address this, over the last decade, we have developed a new optimization algorithm capable of scanning multi-dimensional space and detecting the best solutions (finding the global minimum, as well as the set of local minima). Termed iterative stochastic elimination (ISE), it has been applied to solving structure-based problems [53], as well as ligand-based problems [54,55]. ISE uses an algorithm that can efficiently scan a multi-dimensional space in order to detect the best set of solutions. It has been used to solve problems such as positioning protons [56] and predicting side-chain conformations in proteins [57], scanning the conformational space of loops [58], searching the conformational space of cyclic peptides [59] and loops, predicting drug-likeness and indexing chemicals for their hERG liability [44,55].

Here, we report on the use of the ISE algorithm to construct a model for indexing natural products for their potential anti-diabetic activity and for mapping their discriminative physicochemical properties. Anti-diabetic drugs may act through numerous mechanisms of action and bind with different biological targets. However, as demonstrated by our previous experience [46], the proposed filters-based indexing approach can deal effectively with such complex problems. For this study, we have chosen to screen a database of natural products because they are secondary metabolites of organisms, which means they have been optimized to interact with biological systems through a long natural selection process [60,61,62], and thus, they serve as good drug candidates. It is worth noting that anti-diabetic drug candidates are molecules that, according to the model, have a high chance of exhibiting anti-diabetic activity, but they should be validated using in vivo methods in order to be considered truly anti-diabetic.

## 2. Results and Discussion

### 2.1. Utilization of the Iterative Stochastic Elimination Algorithm for Indexing Natural Compounds for Their Potential Anti-Diabetic Drug Likeness

**Figure 1:**
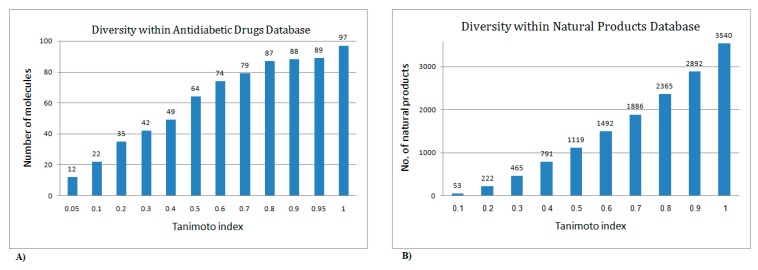
Diversity within anti-diabetic drugs (**A**) and diversity within the natural products database (**B**).

In this study, we used the ISE algorithm to construct a model for indexing natural products for their potential anti-diabetic activity and to map their discriminative physicochemical properties. For the scanning, we used a set of 97 anti-diabetic drugs (presented in the simplified input line-entry system (SMILES) format in the Supporting Information (Table S1)) to represent the active domain. A database composed of 2892 natural products (which was prepared by collecting phytochemicals isolated from more than 800 different plants and which is distributed worldwide and available for purchase from AnalytiCon Discovery company, Postdam, Germany (www.ac-discovery.com)), sharing intermolecular similarity values <0.9, was used to represent the inactive domain. It is worth noting that the 2892 natural products are putative inactives and probably contain a few active chemicals. In order to guarantee that our active/inactive classes would not be biased by similar structures, we first checked for diversity among the 97 anti-diabetic drugs and the 2892 natural products and found them to be very diverse (see Figure 1A,B).

**Figure 2:**
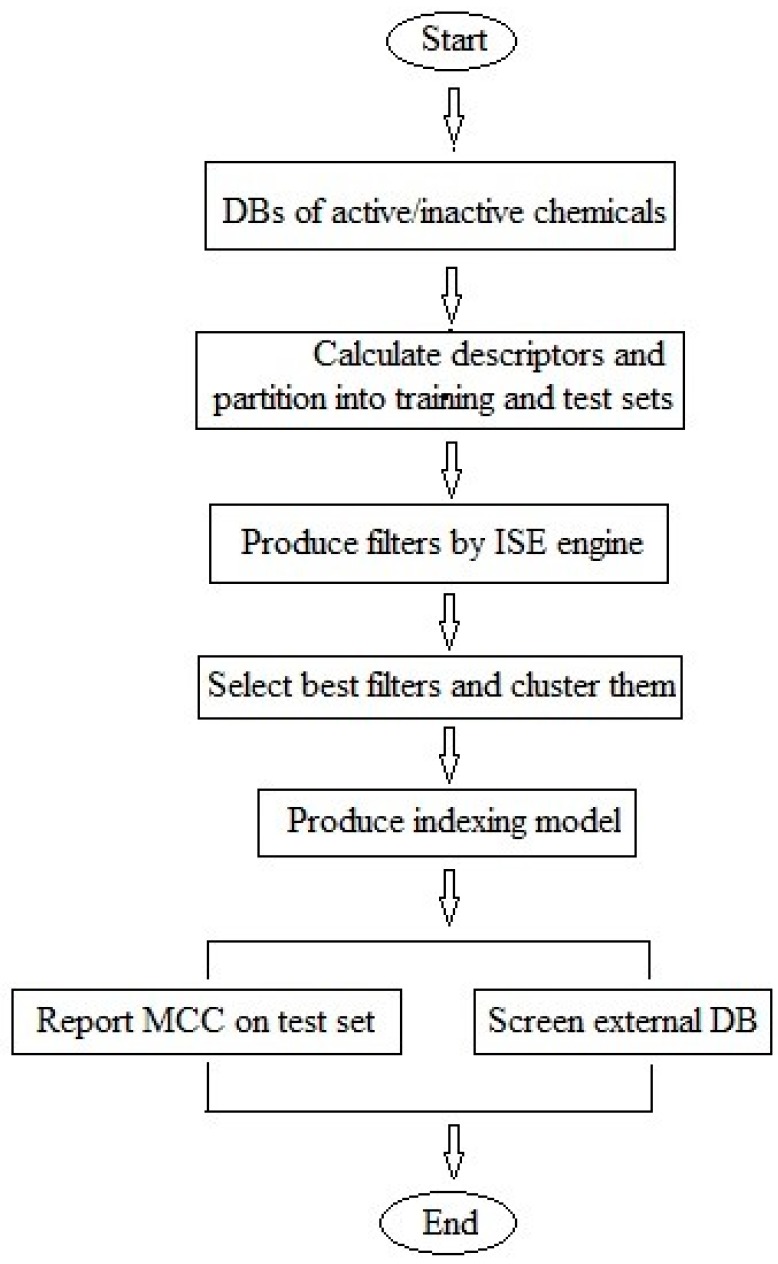
Flowchart for the ligand-based modeling process. ISE, iterative stochastic elimination; MCC, Matthews correlation coefficient.

Optimal differentiation between the active and inactive chemicals was attained by searching in multivariable space for the best sets of descriptors (‘variables’) and the best ranges for all descriptors in each set, capable of distinguishing between actives and inactive chemicals. Since chemical descriptors generally interact with each other, changes in the range of one descriptor could have an effect on the best range of another descriptor, and the optimization process is obliged to take into consideration all descriptors in the set at one time to attain the best set of filters. A flowchart of the modeling process is shown in Figure 2. For further details on applying ISE to obtain the best ranges for a set of descriptors and for the optimization process, see our previously-reported studies [46,55].

### 2.2. Mapping the Discriminative Physicochemical Properties Responsible for Anti-Diabetic Activity

**Figure 3:**
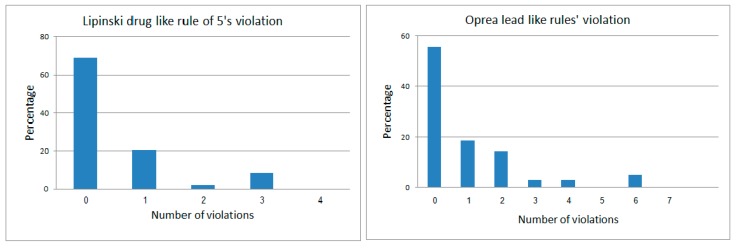
Violation distribution of anti-diabetic drugs for Lipinski’s rule of drug-likeness and Oprea’s rule for lead likeness.

More than 65% of the anti-diabetic drugs had an intermolecular Tanimoto index of similarity <0.7. It is interesting to note that 89.7% of the anti-diabetic drugs obey Lipinski’s rule of five (ROF) [63], and 74.2% obey Oprea’s rule for lead-likeness [64] (see Figure 3).

**Figure 4:**
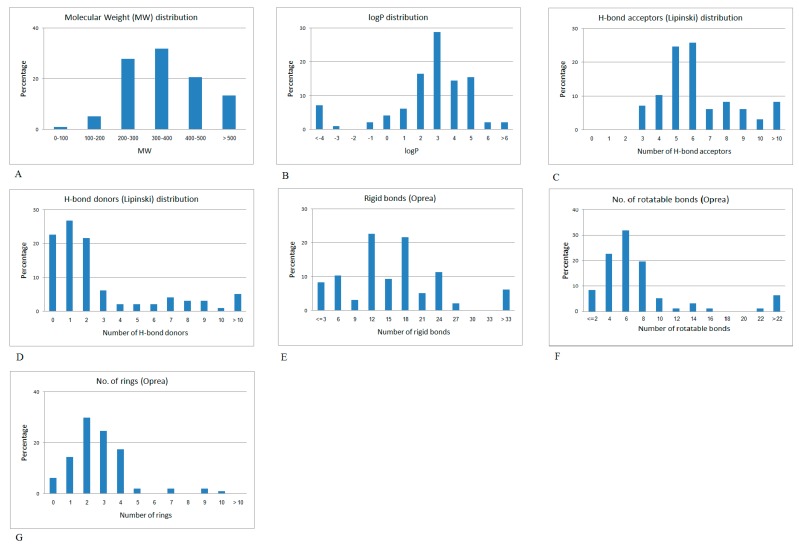
Physicochemical property distribution of anti-diabetic drugs. (**A**) Molecular weight distribution; (**B**) logP values; (**C**) the number of H-bond acceptors (lip_acc as coded by MOE software); (**D**) the number of H-bond donors (lip_don as coded by MOE software); (**E**) the number of rigid bonds; (**F**) the number of rotatable bonds; and (**G**) the number of aromatic atoms.

Figure 4 shows the distribution plots of the Lipinski and Oprea physico-chemical properties of the set of anti-diabetic drugs.

### 2.3. Filters and Descriptors Produced for Constructing the Predictive Model for Indexing Chemicals

To construct the indexing model, we used the ISE algorithm to produce 47 unique filters, composed of different sets and ranges of descriptors. As examples, three such filters are described in Table 1.

The efficiency of the three filters, in terms of their Matthews correlation coefficients, (MCCs), was very close, but they differed in their true positive and negative percentages, as well as in the index attached to each molecule. Filter 2 in Table 1 has an MCC of 0.64, and it identified successfully nearly 63% of the anti-diabetic drugs (true positives), while only 2.77% of the natural products database (presumably inactive) was misclassified (namely, turned up as false positives).

An analysis of the composition of the best filters disclosed a list of discriminative descriptors and/or physico-chemical properties. The data shown in Table 2 describe the most dominant descriptors in the 47 filters used to produce the anti-diabetic activity indexing model. The third column describes how many times the appearances of the descriptor compared to random.

**Figure 5:**
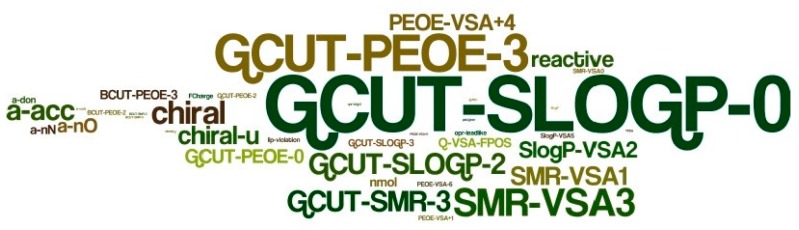
Appearances of descriptors in the 47 filters used to produce the anti-diabetic activity indexing model.

Furthermore, Figure 5 was constructed by utilizing the WORDLE module (a tool for generating "word clouds" from text) and shows the number of appearances of descriptors in a graphic manner. For a full list of all of the descriptors used in the modeling process and their redundancy within the selected best filters, see the Supporting Information (Table S2) in the Appendix. The most dominant descriptors may be valuable for discriminating between anti-diabetic chemicals and inactive ones.

### 2.4. Assessing the Quality of Anti-Diabetic Activity-Indexing Model Potential

**Figure 6:**
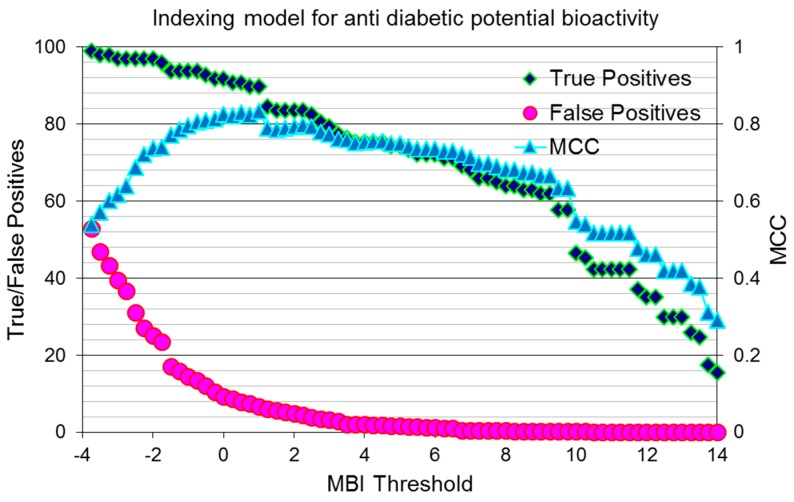
Indexing model for potential anti-diabetic activity: true/false positive percentage (left *y*-axis) and MCCs (right *y*-axis) plotted against the molecular bioactivity index (MBI) threshold (*x*-axis).

To assess the quality of the indexing model, based on the 47 range-based filters, various parameters such as the enrichment factor, Matthews’s correlation coefficient (MCC), ROC curve and the area under the ROC curve (AUC) were generated. The percentage of true/false positives (left *y*-axis) and MCCs (right *y*-axis) were plotted against the molecular bioactivity index (MBI) threshold (*x*-axis), and the results are shown in Figure 6.

**Figure 7:**
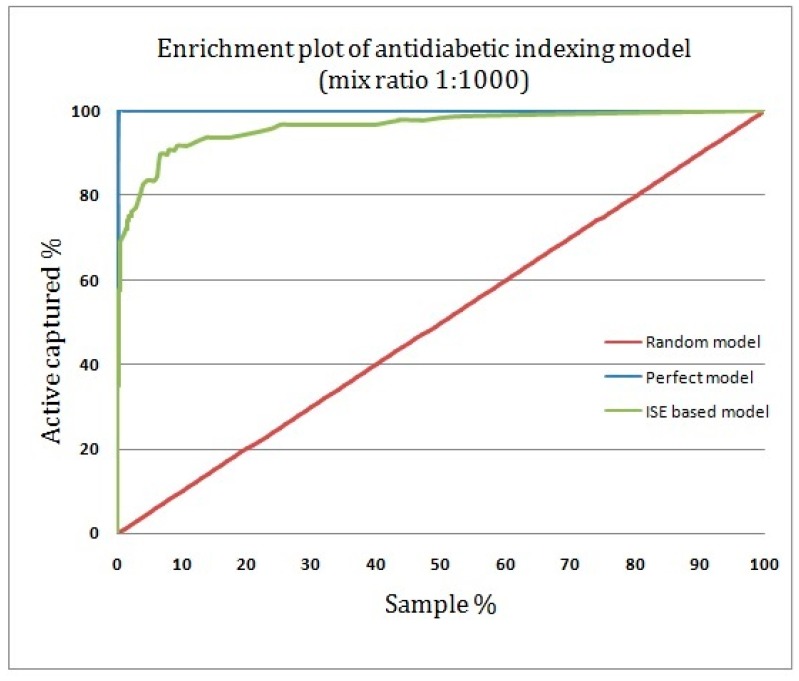
Enrichment plot of the anti-diabetic activity-indexing model.

To illustrate how anti-diabetic drug candidates can be identified if natural products are sorted according to the model’s predictions, rather than based on random selection, an enrichment plot was generated, and it is shown in Figure 7. An enrichment plot of the ISE-based model showing near-perfect results very close to the perfect model at the top fraction indicates a high prioritization power for the proposed model.

**Figure 8:**
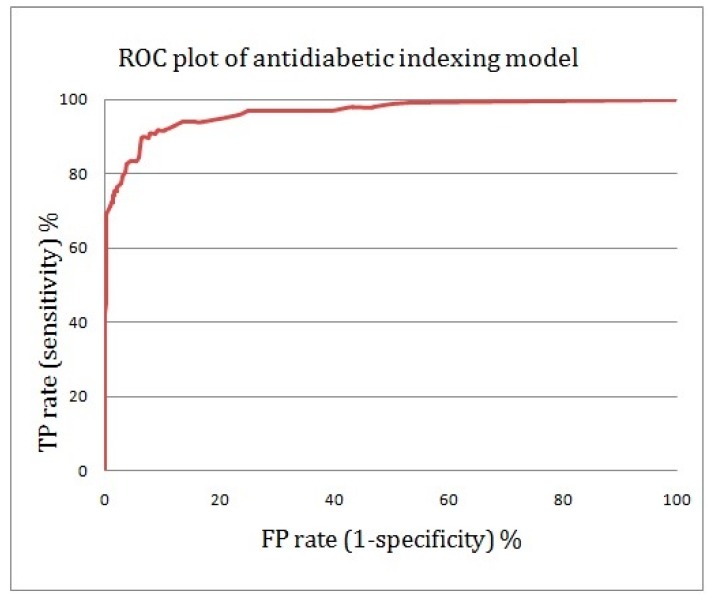
A receiver operating characteristic (ROC) curve showing the performance of the anti-diabetic activity-indexing model (the true positive rate is plotted against the false positive rate).

With the use of the proposed anti-diabetic activity indexing model and a mix ratio of 1:1000 for active to inactive compounds, 65% of the anti-diabetic drugs were captured in the top 1% of the screened compounds, compared to 100% in the perfect model and 1% in the random model. This means that the enrichment factor at the top fraction of 1% is 65-fold (Figure 8).

If we select molecules with an MBI above 13.0, the ISE-based model and the perfect model totally overlap. Thus, it seems that the proposed model is highly discriminative and exhibits very good performance for the classification of anti-diabetic drug candidates versus inactive natural products. The area under the curve (AUC) attained was slightly above 0.96, which indicates that the model is very good.

### 2.5. New Potential Anti-Diabetic Drug Candidates as Disclosed by the ISE-Indexing Model

**Figure 9:**
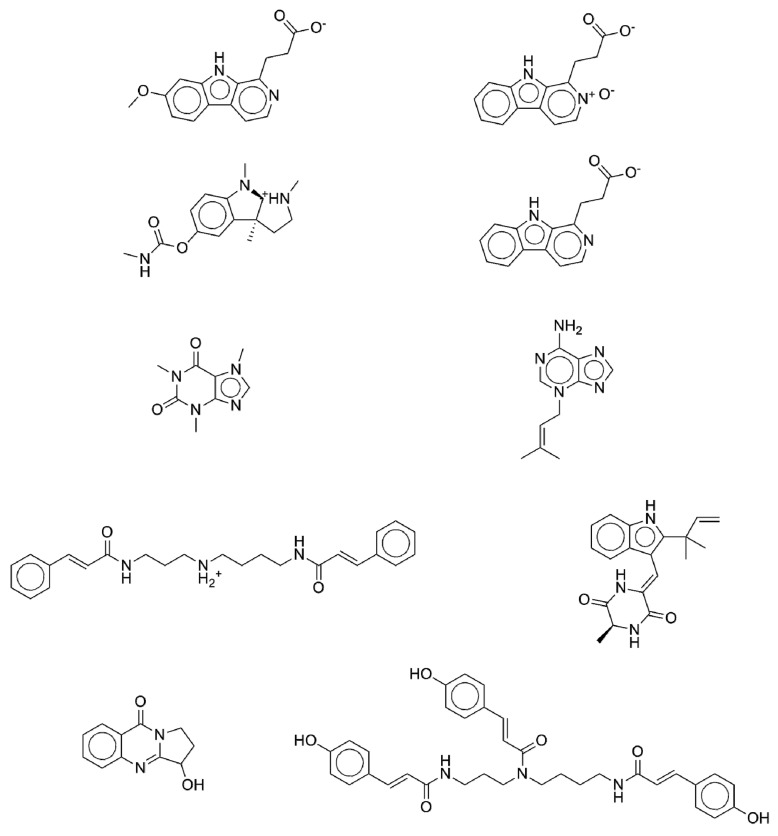
Some of the natural products that scored highly as potential anti-diabetic drug candidates according to our ISE-based, anti-diabetic activity indexing model.

The database composed of 2892 natural products was virtually screened using the aforementioned filter-based indexing model. We assume that few chemicals in the database are anti-diabetic and will get a high MBI score. The MBI score, as shown in Figure 6, ranges between −4.0 (the lowest score) and 14.0 (the highest score). Figure 9 shows ten natural products that scored highly as potential anti-diabetic drug candidates according to our ISE-based anti-diabetic activity indexing model (with MBI score above 8.0). Using the threshold of MBI 8.0, the ratio of TP: FP is equal to 185:1. A search on PubMed revealed that one of the highly indexed phytochemicals (caffeine) has already been tested in vivo and found to be capable of decreasing blood glucose levels, with confirmed anti-diabetic activity [66,67,68]. The other nine phytochemicals await evaluation in the wet lab to ascertain their potential anti-diabetic activity.

## 3. Materials and Methods

For modeling purposes, we used a set of 97 anti-diabetic drugs (presented in SMILES format in the Supporting Information (Table S1)) to represent the active domain. Diversity within the anti-diabetic drugs is shown in Figure 1A. Furthermore, a set composed of 2892 natural products, sharing intermolecular similarity values <0.9, was used to represent the inactive domain. This is justified, since the prediction's model used for virtual screening should cover the properties of chemicals from the screened database, and this database, which was prepared by collecting phytochemicals isolated from more than 800 different plants, is distributed worldwide and is available for purchase from AnalytiCon Discovery company that is located in Postdam, Germany (www.ac-discovery.com). The diversity among the natural products is shown in Figure 1B. It should be noted that the 2892 natural products are putatively inactive and probably contain few active chemical. The usage of a putative inactive set of chemicals that might be ‘contaminated’ with a few active chemicals is discussed in our drug-likeness paper [46]. The ACD database was used as a representative set of non-drugs, although it contains about 1% drugs. The filter-based prediction technique was found to be less sensitive to the ‘contamination’ of the inactive set by 1–2% active compounds.

The physico-chemical properties of all of the chemicals in both databases were calculated using Molecular Operating Environment (MOE) software, Version 2009.10 (http://www.chemcomp.com). The 2D descriptors were based on calculated properties such as molecular weight, log P, H-bond donors/acceptors, solubility, total charge and charge distribution, the types and number of atoms, and so forth [65]. To assess and validate the predictability of the model, the datasets of active/inactive ligands were split into 66.7% for the training set and 33.3% for the test set; an in-house random picking module generated these sets.

The iterative stochastic elimination (ISE) algorithm [46] was implemented to construct models tailored to indexing natural products for potential anti-diabetic activity. Optimal differentiation between active and inactive chemicals was attained by searching, in multivariable space, for the best sets of descriptors (‘variables’) and the best ranges for all descriptors in each set, capable of distinguishing between active and inactive chemicals. Since chemical descriptors generally interact with each other, changes in the range of one descriptor could have an effect on the best range of another descriptor, and the optimization process is obliged to take into consideration all descriptors in the set at one time to attain the best set of filters. The flowchart of the modeling process is shown in Figure 2. For further details on applying the ISE algorithm to obtain the best ranges for a set of descriptors, and for the optimization process, see our previously-reported studies [46,55].

Employing a ‘combined filters approach’ increases the discrimination power and makes it possible to attach to each chemical a molecular bioactivity index (MBI) that correlates with the chance of a molecule’s being bioactive. The MBI concept is based on the assumption that a bioactive ligand would pass more ‘filters’, while an inactive molecule would pass a minimal number of filters. This is the basis for the construction of the MBI index, which is composed of the contribution of the number of filters passed by a molecule to that molecule’s overall quality of potential bioactivity.

(1) $$MBI=\frac{\sum_{i=1}^{n}(\delta Ai\frac{PAi}{PNAi}-\;\delta NAi\frac{NNAi}{NAi})\;}{n}$$

The value of the delta function *δAi* is 0 (zero) if the molecule is inactive according to the currently calculated filter *i* and 1 if it is bioactive according to that filter. Similarly, the value of the delta function *δ_NAi_* is 1 if it is inactive according to filter *i*, and 0 if it is bioactive according to that filter. *PAi* is the percentage of bioactive molecules that are predicted to be ‘bioactives’ according to filter i (‘true positives’), while *PNAi* is the percentage of false positives, i.e., inactive molecules that are predicted to be bioactives according to filter *i*. *NAi* is the percent of bioactives identified to be inactives according to the current filter (‘false negatives’), and N_NAi_ is the percent of inactives identified as such by the current filter (i.e., ‘true negatives’). The quotient *PAi/PNAi* may be regarded as an ‘efficiency factor’ of filter *i* for bioactives, while the quotient *NAi/NNAi* is an ‘efficiency factor’ for misidentifying inactives.

Various parameters such as the enrichment factor, Matthews correlation coefficient (MCC), the ROC curve and the area under ROC curve (AUC) were used to assess the quality of the prediction model. In the ROC curve, sensitivity (the true positive rate) is plotted as a function of the false positive rate. The AUC (area under the ROC curve) is a measure of how well the prediction model can distinguish between two chemicals, one active and one inactive.

## 4. Conclusions

Since diabetes mellitus (DM) is still a leading disease with lethal concomitant complications affecting people worldwide and is associated with an unmet need for developing novel anti-diabetic drugs, several research groups in academia and industry are using in silico techniques to facilitate the discovery of novel anti-diabetic drug candidates, while aiming to save time and costs. We have constructed a prediction model using a set of 97 approved anti-diabetic drugs to represent the active domain and a set of 2892 natural products to represent the inactive domain. It is worth noting that only a few out of the 2892 natural products could have been expected to be anti-diabetic, but the effect of that assumption on the quality of the prediction model is assumed to be negligible. To obtain accurate predictive models for virtual screening purposes, the modeling process should use sets of chemicals that cover the space of the properties of the objects in the screened database. Consequently, we had to select, as the inactive set, objects with the same ‘property space’ as the screened objects. The optimization technique used in this study to index natural products for their potential anti-diabetic bioactivity was the iterative stochastic elimination algorithm. A highly discriminative and robust model was obtained with the area under the curve (AUC) >0.96, indicating a very good prediction model. Upon application of the proposed anti-diabetic activity indexing model to the virtual screening of a set of chemicals with a mix ratio of 1:1000 active-to-inactive compounds, 65% of the anti-diabetic drugs were captured in the top one percent of the screened compounds, compared to 1% in the random model. Some of the natural products that got a high score as anti-diabetic drug candidates are disclosed and presented in Figure 9. A search of the literature revealed that one of the high-scoring phytochemicals (caffeine) has already been tested and reported as an active anti-diabetic molecule, capable to decrease blood glucose levels. The other nine phytochemicals await evaluation in the wet lab for their anti-diabetic activity.

## Figures and Tables

**Table 1:** Efficiency and descriptor ranges of three of the 47 filters used to produce the anti-diabetic activity indexing model.

Filter 1	Filter 2	Filter 3
MCC = 0.642	MCC = 0.640	MCC = 0.638
TP = 86.59%	TP = 62.88%	TP = 81.4%
TN = 77.42%	TN = 97.23%	TN = 82.3%
a_n O (0–6.99)	BCUT_PEOE_2 (0–0.67)	SMR_VSA3 (0–37.82)
PEOE_VSA + 4 (0–30.95)	GCUT_SLOGP_2 (0.11–0.27)	SMR_VSA1 (0–94.72)
Chiral (0–4.99)	Chiral_u (0–3)	GCUT_PEOE_3 (0–2.88)
SlogP_VSA2 (0–65.17)	GCUT_SLOGP_0 (−2.26–−0.91)	Reactive (0–0.00)

NOTE: The efficiency of the filters, in terms of their MCCs, is very close, but they differ in their true positive and negative percentages. In addition, the filters could be composed of different sets and/or ranges of descriptors. The name of descriptors stated herein are the same as named by CCG's computational suite MOE. A full description and methods of descriptors' calculation could be found in the site of Chemical Computing Group [65].

**Table 2:** Number of appearances of descriptors within the set of the best unique filters. The full list of descriptors is presented in the Supporting Information (Table S2). Definition for descriptors and methods of its calculation could be found in the site if Chemical Computing Group [65].

Descriptor Name	No. of Appearances	Redundant More Times than Random
GCUT_SLOGP_0	24	23.7
a_ICM	16	15.8
PEOE_VSA + 4	12	11.9
SMR_VSA1	10	9.9
logS	9	8.9
Nmol	9	8.9
lip_druglike	9	8.9
Chi1_C	8	7.9
GCUT_PEOE_0	8	7.9
opr_leadlike	7	6.9
Q_VSA_FPOS	7	6.9
SMR_VSA3	7	6.9
a_don	6	5.9
a_hyd	6	5.9

## Supplementary Materials

The following are available online. Table S1 listing the 97 anti-diabetic drugs. Table S2 including a full list of all of the descriptors used in the modeling process and their redundancy within the selected best filters.
