# Supplementary material for: Indexing Natural Products for Their Potential Anti-Diabetic Activity: Filtering and Mapping Discriminative Physicochemical Properties
Source: Molecules. 2017 Sep 17;22(9):1563. doi: 10.3390/molecules22091563 (PMC6151781; doi:10.3390/molecules22091563)
Supplement: Supplementary file 1 [file molecules-22-01563-s001.pdf]

SI table 1: List of ninety seven anti-diabetic drugs.

```
c1([S@@](NC(NCCCC)=O)(=O)=O)ccc(C)cc1    TOLBUTAMIDE
c1([S](Nc2sc([CH](C)C)nn2)(=O)=O)ccc(N)cc1    GLYPROTHIAZOL
c1([S](NC(NCCC)=O)(=O)=O)ccc(Cl)cc1    CHLORPROPAMIDE
c1(CCNC(NC(N)=N)=N)ccccc1    PHENFORMIN
c1([S@@](NC(NCCCC)=O)(=O)=O)ccc(N)cc1    CARBUTAMIDE
c1([S@@](Nc2ncc(OCCOC)cn2)(=O)=O)ccccc1 GLYMIDINE
c12c(ccc(c1)[S](NC(N[CH]1CCCCC1)=O)(=O)=O)CCC2    GLYHEXAMIDE
c1(sc([C](C)(C)C)nn1)N[S](c1ccc(N)cc1)(=O)=O  GLYBUTHIAZOL
c1([S](NC(N[CH]2CCCCC2)=O)(=O)=O)cc(c(C)cc1)N METAHEXAMIDE
c1([S](NC(N[N]2CCCC2)=O)(=O)=O)ccc(Cl)cc1    GLYCLOPYRAMIDE
C(NC(N)=N)([N](C)C)=N  METFORMIN
c1([S](NC(N[CH]2CCCCC2)=O)(=O)=O)ccc(C)cc1    GLYCYCLAMIDE
N(C(NC(N)=N)=N)CCCC    BUFORMIN
c1([S](NC(N[CH]2CCCCC2)=O)(=O)=O)ccc(C(C)=O)cc1    ACETOHEXAMIDE
c1([S](NC(N[CH]2CCCC2)=O)(=O)=O)ccc(C)cc1    TOLPENTAMIDE
c1([S](NC(N[CH]2CCCCCC2)=O)(=O)=O)ccc(C)cc1   HEPTOLAMIDE
c1([S](NC(N[CH]2CCCCCCC2)=O)(=O)=O)ccc(C)cc1  GLYOCTAMIDE
c1([S](NC(N[N]2CCCCCC2)=O)(=O)=O)ccc(C)cc1    TOLAZAMIDE
c1([S](NC(N[N]2CCCCCC2)=O)(=O)=O)ccc(Cl)cc1   GLYPINAMIDE
c1(sc([C](C)(C)C)nn1)N[S](c1ccccc1)(=O)=O    GLYBUZOLE
c1(c(ccc(c1)OC)OC)[CH]([CH](N[C](C)(C)C)C)O   BUTOXAMINE
c12c(ccc(c1)[S](NC(N[N]1CCCCCC1)=O)(=O)=O)CCC2    GLIDAZAMIDE
c1([S](Nc2sc(C[CH](C)C)nn2)(=O)=O)ccc(OC)cc1  GLYSOBUZOLE
c1([S](NC(N[CH]2CCCCC2)=O)(=O)=O)ccc(SC)cc1   THIOHEXAMIDE
C(CCCNC(N)=N)(N)=O    TIFORMIN
c1(ccc([N](C)C)cc1)NC(N[S](c1ccc(Cl)cc1)(=O)=O)=O   GLYPARAMIDE
c1([S@@](NC([N]2CCCC2)=O)(=O)=O)ccc(C)cc1    TOLPYRRAMIDE
c1([S](Nc2sc(CCCC)nn2)(=O)=O)ccc(Cl)cc1 BUTADIAZAMIDE
c1(c(ccc(c1)Cl)OC)C(NCCc1ccc([S](NC(N[CH]2CCCCC2)=O)(=O)=O)cc1)=O
      GLYBURIDE
c1([S](=O)(=O)[O-])c(ccc(c1)O)O.c1([S](=O)(=O)[O-])c(ccc(c1)O)O.[Ca+2]
      CALCIUM DOBESILATE
C1[CH]2[CH](C[N]1NC(N[S](c1ccc(C)cc1)(=O)=O)=O)CCC2 GLICLAZIDE
c1(c(ccc(c1)Cl)OC)NC(Cc1ccc([S](Nc2ncc(C[CH](C)C)cn2)(=O)=O)cc1)=O
      GLICETANILE
c1(ccc(CCNC(c2noc(c2)C)=O)cc1)[S](NC(N[CH]1CCCCC1)=O)(=O)=O
      GLISOLAMIDE
c1(ccc(CCNC(c2noc(c2)C)=O)cc1)[S](NC(N[N]1CCCCCC1)=O)(=O)=O
      GLISOXEPIDE
C1([N]([CH]2CCC=CC2)CC[N]1[S](c1ccc(CCNC(CCC)=O)cc1)(=O)=O)=N
      GLIBUTIMINE
C1[C]2([C]([C@H]([C@@H]([C@@H]2O)NC(N[S@@](c2ccc(C)cc2)(=O)=O)=O)(C1))(C)
C)C   GLIBORNURIDE
c1(ccc(CCNC(c2cnc(C)cn2)=O)cc1)[S](NC(N[CH]1CCCCC1)=O)(=O)=O   GLIPIZIDE
c1(c(cccc1)OC)C(NCCc1ccc([S@@](NC(N[CH]2CCCC2)=O)(=O)=O)cc1)=O GLIPENTIDE
c12c(C([N](CCc3ccc([S](NC(N[CH]4CCCCC4)=O)(=O)=O)cc3)C([C]1(C)C)=O)=O)cc(
OC)cc2    GLIQUIDONE
c1(c(ccc(c1)F)OC)[C@H](NC(Cc1ccc([S](Nc2ncc(C[CH](C)C)cn2)(=O)=O)cc1)=O)(
C)   GLIFLUMIDE
c12c([n](CC)nc2C)ncc(c1OCC[CH](C)C)C(NCCc1ccc([S](NC(N[CH]2CCCCC2)=O)(=O)
=O)cc1)=O   GLICARAMIDE
C(/NCCCC)(NC(N)=N)=N/CC    ETOFORMIN
```

C1[CH]2[CH](C[CH]1C=C2)CNC(N[S]([N]1CC[CH](CCNC(c2c(nccc2)OC)=O)CC1)(=O)=O)=O GLIAMILIDE
c1([S](NC(N[CH]2CC[CH](C)CC2)=O)(=O)=O)ccc(CCNC([N](c2ccccn2)C)=O)cc1 GLISAMURIDE
c1(c(ccc(c1)Cl)OC)C(NCCc1ccc([S](NC(NC)=O)(=O)=O)cc1)=O GLICONDAMIDE
c1(cc(c(N)cc1)C#N)[CH](CN[CH](C)C)O CIMATEROL
c1(c(ccc(c1)Cl)OC)C(NCCc1ccc(C(O)=O)cc1)=O MEGLITINIDE
C([N]1CCCC1)(/N=C1\[N](CCC1)C)=N\c1ccccc1 PIROGLIRIDE
c1([CH](c2ccccn2)CC=2NCCN2)ccccc1 MIDAGLIZOLE
C1[C](O1)(CCCCCCCCCCCCCC)C(O)=O PALMOXIRIC ACID
C1[C](O1)(CCCCCCCCCCCCCC)C(OC)=O METHYL PALMOXIRATE
c12c(C[N](C1=O)C(NCCc1ccc([S@@](NC(N[CH]3CCCCC3)=O)(=O)=O)cc1)=O)cccc2 GLISINDAMIDE
C1[N]([C@@H]([C@H]([C@@H](O)[C@H]1O)O)CO)CCO MIGLITOL
C([CH]1SC(=O)NC1=O)c1ccc(OC[C]2(CCCCC2)C)cc1 CIGLITAZONE
C([N]1CCOCC1)(/N=C1\[N](CCC1)C)=N\c1ccccc1 LINOGLIRIDE
c1(c2c(cccc2)[nH]c1)C[C@H]1NC([C@H](Cc2ccc(O)cc2)NC([C@@H]([N@](C([C@H](Cc2ccccc2)NC([C@@H](NC([C@@H](NC1=O)CCCCN)=O)[CH](C)C)=O)=O)C)C)=O)=O SEGLITIDE
C1(/C([N](CC(O)=O)C(S1)=S)=O)=C\C(=C/c1ccccc1)C EPALRESTAT
C1[C](O1)(CCCCCc1ccc(Cl)cc1)C(O)=O CLOMOXIR
c12[C]3(c4c(ccc(c4)F)c1ccc(c2)F)C(NC(N3)=O)=O IMIRESTAT
C1([N](CC(C)=C1CC)C(NCCc1ccc([S](NC(N[CH]2CC[CH](C)CC2)=O)(=O)=O)cc1)=O)=O GLIMEPIRIDE
C([CH]1C(NC(S1)=O)=O)c1ccc(OCCc2ccc(CC)cn2)cc1 PIOGLITAZONE
c12c(C[N](C1)NC=1NCCN1)cccc2F ISAGLIDOLE
c12c(c([n](Cc3nc4cc([C@@](F)(F)F)ccc4s3)nc1CC(O)=O)=O)cccc2 ZOPOLRESTAT
C(NN)(N)=N PIMAGEDINE
C1[C@@H]([C@@H]([C@@H](O)[C@@H]([C@]1(CO)O)O)O)N[CH](CO)CO VOGLIBOSE
c12c(CC[C](O1)(COc1ccc(C[CH]3C(NC(S3)=O)=O)cc1)C)c(c(O)c(c2C)C)C TROGLITAZONE
c12c(O[CH](Cc3ccccc3)CC2)ccc(c1)C[CH]1C([N]([Na])C(S1)=O)=O ENGLITAZONE
C=1([C]2([N]3c4c(cccc4C2)CC3)CCC)NCCN1 DERIGLIDOLE
C([N]1[C@@H]([C@H]([C@H](O)[C@H](C1)O)O)CO)[C@H]1O[C@@H]([C@H](O)[C@H]([C@@H]1O)O)OC CAMIGLIBOSE
c1(c2ccccc2)nc(CCC(c2ccc(C[CH]3C([N]([Na])C(S3)=O)=O)cc2)=O)c(o1)C DARGLITAZONE
c1(CNC(/N=C(/N[P](=O)([O-])[O-])N)=N)ccccc1.[Na+].[Na+] BENFOSFORMIN
c12c(c[nH]c1cccc2)C[C@@H](C(N[C@H](C(N[C@H](C(N[C@@H](Cc1ccccc1)C(N)=O)=O)CC(O)=O)=O)CCSC)=O)N TETRAGASTRIN
C([C@@H](C(NCCCCCCCC)=O)N)S[V](SC[C@@H](C(NCCCCCCCC)=O)N)=O NAGLIVAN
C([N]1CCCCC1)[CH](CO\N=C(\c1cccnc1)Cl)O BIMOCLOMOL
c1([CH](NCc2cc(ccc2)[C](F)(F)F)C(O)=O)ccccc1 PHENYLGLYCINE-BASED INSULIN SENSITIZER
c12c(C[C@H](NC([C@@H](NC([C@@H](NC([C@H](Cc3ccccc3)NC([C@@H](NC([C@@H](NC([C@@H](NC([C@@H](NC([C@@H](NC([C@@H](NC([C@@H](NC([C@@H](NC([C@H](Cc3ccc(O)cc3)NC([C@@H](NC([C@@H](NC([C@H](Cc3ccc(O)cc3)NC([C@@H](NC([C@@H](NC([C@@H](NC([C@H](Cc3ccccc3)NC([C@@H](NC(CNC([C@@H](NC([C@@H](NC([C@H](Cc3c[nH]cn3)N)=O)CO)=O)CCC(N)=O)=O)=O)[C@@H](C)O)=O)=O)[C@@H](C)O)=O)CO)=O)CC(O)=O)=O)=O)CO)=O)CCCCN)=O)=O)C[CH](C)C)=O)CC(O)=O)=O)CO)=O)CCCNC(N)=N)=O)CCCNC(N)=N)=O)C)=O)CCC(N)=O)=O)CC(O)=O)=O)=O)[CH](C)C)=O)CCC(N)=O)=O)C(N[

C@H](C(N[C@H](C(N[C@H](C(N[C@@H]([C@@H](C)O)C(O)=O)=O)CC(N)=O)=O)CCSC)=O)
C[CH](C)C)=O)c[nH]c1cccc2   GLUCAGON
C1[C@H]2([C@H]([C@H]3([C](C(=O)CC3)(C)C1))(CC=C1[C]2(CC[C@@H](C1)O[S](O)(
=O)=O)C)) 3)- SULFOXY)ANDROST-5-EN-17-ONE
C([CH]1SC(=O)NC1=O)c1ccc(OCC[N](c2ccccn2)C)cc1   ROSIGLITAZONE
C([C@H]1CC[C@H]([C@@H](C)C)CC1)(N[C@H](Cc1ccccc1)C(O)=O)=O
NATEGLINIDE
c12[C]3(C[C@@H](C(N)=O)Oc1ccc(c2)F)NC(=O)NC3=O   FIDARESTAT
c1([S](NC(NCCCC)=O)(=O)=O)ccccc1   PHENBUTAMIDE
c1([S@@](NC(N[N]2CCCCC2)=O)(=O)=O)ccc(Cl)cc1 CHLORPENTAZIDE
C([C@@H]1C(=C)C1)[CH](NC(CC[C@@H](C(O)=O)N)=O)C(O)=O   HYPOGLYCIN B
c1([S](NC(N[CH]2CCCCC2)=O)(=O)=O)ccc(Cl)cc1   CHLORHEXAMIDE
c1([S](NC(N[CH]2CCCCC2)=O)(=O)=O)ccc([CH](C)O)cc1   HYDROXYHEXAMIDE
C1=2[C]3([C]([C@@H]([C]([C@@H](C[C@H]4([C@@H](C(=O)O[C@@H]4C)C))O)(C)O)CC
3)(CC[C@@H]1([C]1([C@@H](C[C@@H](O)[C@H](C1)O)(C(C2)=O))C))C)O CYASTERONE
O([C@@H]1[C@H](O[C@H](O[C@@H]2[C@H](O[C@H](O[C@H]3O[C@@H]([C@@H](O)[C@@H]
([C@H]3O)O)CO)[C@@H]([C@H]2O)O)CO)[C@@H]([C@H]1O)O)CO)[C@H]1O[C@@H]([C@@H
](O[C@@H]2[C@@H]([C@H]([C@H](N[C@H]3C=C([C@@H](O[C@H]4O[C@@H]([C@@H](O[C@
@H]5[C@@H]([C@H]([C@H](N[C@H]6C=C([C@@H](O)[C@@H]([C@H]6O)O)CO)[C@H](O5)C
)O)O)[C@@H]([C@H]4O)O)CO)[C@@H]([C@H]3O)O)CO)[C@H](O2)C)O)O)[C@@H]([C@H]1
O)O)CO   TRESTATIN A
c1(c([C@@H](NC(Cc2cc(c(C(O)=O)cc2)OCC)=O)C[CH](C)C)cccc1)[N]1CCCCC1
REPAGLINIDE
C([N]1[C@@H](CCC1)C(N[C@@H](CCCCNC(CCCCCCCCCCCC)=O)C(O)=O)=O)([C@@H](NC(
[C@H](Cc1ccc(O)cc1)NC([C@H](Cc1ccccc1)NC([C@H](Cc1ccccc1)NC(CNC([C@@H](NC
([C@@H](NC(CNC([C@H]1NC([C@@H](NC([C@@H](NC([C@H](Cc2ccc(O)cc2)NC([C@@H](
NC([C@@H](NC([C@@H](NC([C@@H](NC([C@@H](NC([C@H](Cc2c[nH]cn2)NC([C@@H](NC
(CNC([C@@H](NC([C@@H](NC([C@H](Cc2c[nH]cn2)NC([C@@H](NC([C@@H](NC([C@@H](
NC([C@H](Cc2ccccc2)N)=O)[C@@H](C)C)=O)CC(N)=O)=O)CCC(N)=O)=O)=O)C[CH](C)C
)=O)CSSC[C@@H]2NC([C@H](CSSC[C@@H](C(N[C@H](C(N[C@H](C(N[C@H](C(N[C@H](C(
N[C@H](C(N[C@H](C(N[C@H](C(N[C@H](C(N[C@@H](CSSC1)C(N[C@@H](CC(N)=O)C(O)=
O)=O)=O)Cc1ccc(O)cc1)=O)CC(N)=O)=O)CCC(O)=O)=O)C[CH](C)C)=O)CCC(N)=O)=O)C
c1ccc(O)cc1)=O)C[CH](C)C)=O)CO)=O)NC([C@@H](NC([C@@H](NC([C@@H](NC2=O)[C@
@H](C)O)=O)CO)=O)[C@H](CC)C)=O)NC([C@@H](NC([C@@H](NC([C@@H](NC([C@@H](NC
(CN)=O)[C@H](CC)C)=O)[C@@H](C)C)=O)CCC(O)=O)=O)CCC(N)=O)=O)=O)=O)=O)CO)=O
)=O)C[CH](C)C)=O)[C@@H](C)C)=O)CCC(O)=O)=O)C)=O)C[CH](C)C)=O)=O)C[CH](C)C
)=O)[C@@H](C)C)=O)=O)=O)CCC(O)=O)=O)CCCNC(N)=N)=O)=O)=O)=O)=O)[C@@H](C)O)
=O   MCMC00010636
C([C@@H](C(N[C@@H](Cc1ccc(O)cc1)C(N[C@H](C(N[C@H](C(N[C@H](C(N[C@@H]([C@@
H](C)O)C(O)=O)=O)CCCCN)=O)CC(O)=O)=O)[C@@H](C)O)=O)=O)NC([C@H](Cc1ccccc1)
NC(CNC([C@@H](NC([C@@H](NC(CNC([C@H]1NC([C@@H](NC([C@@H](NC([C@H](Cc2ccc(
O)cc2)NC([C@@H](NC([C@@H](NC([C@@H](NC([C@@H](NC([C@@H](NC([C@H](Cc2c[nH]
cn2)NC([C@@H](NC(CNC([C@@H](NC([C@@H](NC([C@H](Cc2c[nH]cn2)NC([C@@H](NC([
C@@H](NC([C@@H](NC([C@H](Cc2ccccc2)N)=O)[C@@H](C)C)=O)CC(N)=O)=O)CCC(N)=O
)=O)=O)C[CH](C)C)=O)CSSC[C@@H]2NC([C@H](CSSC[C@@H](C(N[C@H](C(N[C@H](C(N[
C@H](C(N[C@H](C(N[C@H](C(N[C@H](C(N[C@H](C(N[C@H](C(N[C@@H](CSSC1)C(N[C@@
H](CC(N)=O)C(O)=O)=O)=O)Cc1ccc(O)cc1)=O)CC(N)=O)=O)CCC(O)=O)=O)C[CH](C)C)
=O)CCC(N)=O)=O)Cc1ccc(O)cc1)=O)C[CH](C)C)=O)CO)=O)NC([C@@H](NC([C@@H](NC(
[C@@H](NC2=O)[C@@H](C)O)=O)CO)=O)[C@H)[CC)C)=O)NC([C@@H](NC([C@@H](NC([C@
@H](NC([C@@H](NC(CN)=O)[C@H](CC)C)=O)[C@@H](C)C)=O)CCC(O)=O)=O)CCC(N)=O)=
O)=O)=O)=O)CO)=O)=O)C[CH](C)C)=O)[C@@H](C)C)=O)CCC(O)=O)=O)C)=O)C[CH](C)C
)=O)=O)C[CH](C)C)=O)[C@@H](C)C)=O)=O)=O)CCC(O)=O)=O)CCCNC(N)=N)=O)=O)=O)c
1ccccc1   MCMC00010665

```
C([C@H]1[N](C([C@@H](NC([C@@H](NC([C@H]2[N](CCC2)C(CNC([C@H](Cc2ccccc2)NC
([C@@H](NC([C@@H](NC([C@@H](NC([C@@H](NC([C@H](Cc2c[nH]cn2)NC([C@@H](NC([
C@@H](NC([C@H](Cc2ccccc2)NC([C@@H](NC([C@@H](NC([C@@H](NC([C@@H](NC([C@@H
](NC([C@@H](NC([C@@H](NC([C@H]2NC([C@@H](NC([C@@H](NC([C@@H](NC([C@@H](NC
([C@H](CSSC2)NC([C@H](CCCCN)N)=O)=O)CC(N)=O)=O)[C@@H](C)O)=O)C)=O)[C@@H](
C)O)=O)=O)C)=O)[C@@H](C)O)=O)CCC(N)=O)=O)CCCNC(N)=N)=O)C[CH](C)C)=O)C)=O)
CC(N)=O)=O)=O)C[CH](C)C)=O)[C@@H](C)C)=O)=O)CO)=O)CO)=O)CC(N)=O)=O)CC(N)=
O)=O)=O)=O)=O)[C@H](CC)C)=O)C[CH](C)C)=O)CCC1)([N]1[C@@H](CCC1)C(N[C@H](C
(N[C@H](C(N[C@H](C(NCC(N[C@H](C(N[C@H](C(N[C@H](C(N[C@@H](Cc1ccc(O)cc1)C(
N)=O)=O)[C@@H](C)O)=O)CC(N)=O)=O)CO)=O)=O)[C@@H](C)C)=O)CC(N)=O)=O)[C@H](
C)O)=O)=O   MCMC00010668
c12c(CC[C@](O1)(COc1ccc(C[C@H]3C(NC(S3)=O)=O)cc1)C)c(c(O)c(c2C)C)C
      )S,S)-TROGLITAZONE
c12c(CC[C@](O1)(COc1ccc(C[C@@H]3C(NC(S3)=O)=O)cc1)C)c(c(O)c(c2C)C)C
      )S,R)-TROGLITAZONE
c12c(CC[C](O1)(COc1ccc(C[C@H]3C(NC(S3)=O)=O)cc1)C)c(c(O)c(c2C)C)C
      )R,S)-TROGLITAZONE
c12c(CC[C](O1)(COc1ccc(C[C@@H]3C(NC(S3)=O)=O)cc1)C)c(c(O)c(c2C)C)C
      )R,R)-TROGLITAZONE
S1C(NC([CH]1Cc1ccc(cc1)OCc1nc2ccccc2c([n]1C)=O)=O)=O      MCMC00010890
c1(ccccc1)C[C@@H](Oc1c(cc(cc1C)c1c2c(sc(c2C)C)c(c2c1cccc2)Br)C)C(=O)O
      MCMC00010916
```

SI_table 2: Full list of all descriptors used in modeling process and their redundancy within the selected best filters.

Descriptor name	Redundancy
GCUT_SLOGP_0	24
a_ICM	16
PEOE_VSA+4	12
SMR_VSA1	10
logS	9
nmol	9
lip_druglike	9
chi1_C	8
GCUT_PEOE_0	8
opr_leadlike	7
Q_VSA_FPOS	7
SMR_VSA3	7
a_don	6
a_hyd	6
BCUT_SMR_3	4
chi1v_C	4
chiral	4
GCUT_PEOE_3	4
GCUT_SLOGP_3	3
PEOE_VSA_NEG	3
SMR_VSA6	2
a_nH	2
a_nN	2
a_nO	2
b_single	2
chi0v_C	2
chiral_u	2
PEOE_PC+	2
PEOE_VSA+5	2
PEOE_VSA_FNEG	1
a_IC	1
BCUT_SLOGP_3	1
bpol	1
b_count	1
b_double	1
chi0_C	1
density	1
GCUT_PEOE_1	1
GCUT_SMR_3	1
lip_violation	0
logP(o/w)	0
Q_VSA_FNEG	0

SI_table 2: Full list of all descriptors used in modeling process and their redundancy within the selected best filters.

SlogP_VSA8	0
SMR_VSA0	0
vsa_hyd	0
apol	0
a_acc	0
a_acid	0
a_aro	0
a_base	0
a_count	0
a_heavy	0
a_nB	0
a_nBr	0
a_nC	0
a_nCl	0
a_nF	0
a_nI	0
a_nP	0
a_nS	0
balabanJ	0
BCUT_PEOE_0	0
BCUT_PEOE_1	0
BCUT_PEOE_2	0
BCUT_PEOE_3	0
BCUT_SLOGP_0	0
BCUT_SLOGP_1	0
BCUT_SLOGP_2	0
BCUT_SMR_0	0
BCUT_SMR_1	0
BCUT_SMR_2	0
b_1rotN	0
b_1rotR	0
b_ar	0
b_heavy	0
b_rotN	0
b_rotR	0
b_triple	0
chi0	0
chi0v	0
chi1	0
chi1v	0

SI_table 2: Full list of all descriptors used in modeling process and their redundancy within the selected best filters.

diameter	0
FCharge	0
GCUT_PEOE_2	0
GCUT_SLOGP_1	0
GCUT_SLOGP_2	0
GCUT_SMR_0	0
GCUT_SMR_1	0
GCUT_SMR_2	0
Kier1	0
Kier2	0
Kier3	0
KierA1	0
KierA2	0
KierA3	0
KierFlex	0
lip_acc	0
lip_don	0
mr	0
mutagenic	0
opr_brigid	0
opr_nring	0
opr_nrot	0
opr_violation	0
PC+	0
PC-	0
PEOE_PC-	0
PEOE_RPC+	0
PEOE_RPC-	0
PEOE_VSA+0	0
PEOE_VSA+1	0
PEOE_VSA+2	0
PEOE_VSA+3	0
PEOE_VSA+6	0
PEOE_VSA-0	0
PEOE_VSA-1	0
PEOE_VSA-2	0
PEOE_VSA-3	0
PEOE_VSA-4	0
PEOE_VSA-5	0
PEOE_VSA-6	0

SI_table 2: Full list of all descriptors used in modeling process and their redundancy within the selected best filters.

PEOE_VSA_FHYD	0
PEOE_VSA_FPNEG	0
PEOE_VSA_FPOL	0
PEOE_VSA_FPOS	0
PEOE_VSA_FPPOS	0
PEOE_VSA_HYD	0
PEOE_VSA_PNEG	0
PEOE_VSA_POL	0
PEOE_VSA_POS	0
PEOE_VSA_PPOS	0
petitjean	0
petitjeanSC	0
Q_PC+	0
Q_PC-	0
Q_RPC+	0
Q_RPC-	0
Q_VSA_FHYD	0
Q_VSA_FPNEG	0
Q_VSA_FPOL	0
Q_VSA_FPPOS	0
Q_VSA_HYD	0
Q_VSA_NEG	0
Q_VSA_PNEG	0
Q_VSA_POL	0
Q_VSA_POS	0
Q_VSA_PPOS	0
radius	0
reactive	0
rings	0
RPC+	0
RPC-	0
rsynth	0
SlogP	0
SlogP_VSA0	0
SlogP_VSA1	0
SlogP_VSA2	0
SlogP_VSA3	0
SlogP_VSA4	0
SlogP_VSA5	0
SlogP_VSA6	0

SI_table 2: Full list of all descriptors used in modeling process and their redundancy within the selected best filters.

SlogP_VSA7	0
SlogP_VSA9	0
SMR	0
SMR_VSA2	0
SMR_VSA4	0
SMR_VSA5	0
SMR_VSA7	0
TPSA	0
VAdjEq	0
VAdjMa	0
VDistEq	0
VDistMa	0
vdw_area	0
vdw_vol	0
vsa_acc	0
vsa_acid	0
vsa_base	0
vsa_don	0
vsa_other	0
vsa_pol	0
Weight	0
weinerPath	0
weinerPol	0
zagreb	0
